# Colchicine ameliorates myocardial injury induced by coronary microembolization through suppressing pyroptosis via the AMPK/SIRT1/NLRP3 signaling pathway

**DOI:** 10.1186/s12872-023-03697-8

**Published:** 2024-01-03

**Authors:** Hongqing Li, Huafeng Yang, Zhenbai Qin, Qiang Wang, Lang Li

**Affiliations:** 1 Department of Cardiology, The First Affiliated Hospital of Guangxi Medical University, Guangxi Cardiovascular Institute, Nanning, Guangxi 530021 China; 2 Cardiothoracic Surgery Intensive Care Unit, The First Affiliated Hospital of Guangxi Medical University, Nanning, Guangxi China

**Keywords:** Coronary microembolization, Colchicine, Pyroptosis, AMPK/SIRT1/NLRP3

## Abstract

**Background:**

Coronary microembolization(CME)is a common complication in acute coronary syndrome and percutaneous coronary intervention, which is closely related to poor prognosis. Pyroptosis, as an inflammatory programmed cell death, has been found to be associated with CME-induced myocardial injury. Colchicine (COL) has potential benefits in coronary artery disease due to its anti-inflammatory effect. However, the role of colchicine in pyroptosis-related CME-induced cardiomyocyte injury is unclear. This study was carried out to explore the effects and mechanisms of colchicine on myocardial pyroptosis induced by CME.

**Methods:**

The CME animal model was constructed by injecting microspheres into the left ventricle with Sprague-Dawley rats, and colchicine (0.3 mg/kg) pretreatment seven days before and on the day of modeling or compound C(CC)co-treatment was given half an hour before modeling. The study was divided into 4 groups: Sham group, CME group, CME + COL group, and CME + COL + CC group (10 rats for each group). Cardiac function, serum myocardial injury markers, myocardial histopathology, and pyroptosis-related indicators were used to evaluate the effects of colchicine.

**Results:**

Colchicine pretreatment improved cardiac dysfunction and reduced myocardial injury induced by CME. The main manifestations were the improvement of left ventricular systolic function, the decrease of microinfarction area, and the decrease of mRNA and protein indexes related to pyroptosis. Mechanistically, colchicine increased the phosphorylation level of adenosine monophosphate-activated protein kinase (AMPK), promoted the expression of silent information regulation T1 (SIRT1), and inhibited the expression of NOD-like receptor pyrin containing 3 (NLRP3) to reduce myocardial pyroptosis. However, after CC co-treatment with COL, the effect of colchicine was partially reversed.

**Conclusion:**

Colchicine improves CME-induced cardiac dysfunction and myocardial injury by inhibiting cardiomyocyte pyroptosis through the AMPK/SIRT1/NLRP3 signaling pathway.

**Supplementary Information:**

The online version contains supplementary material available at 10.1186/s12872-023-03697-8.

## Introduction

Coronary microembolization (CME) is initially observed at autopsy and postmortem angiography in patients with ischemic heart disease. Studies have found that CME occurred not only during spontaneous plaque rupture and ulceration, but also during coronary interventions. The occurrence of CME is associated with a decrease in coronary flow reserve, progressive myocardial systolic dysfunction, and perfusion contractile function mismatch [1, 2]. For the prevention and treatment of CME, the routine use of interventional protective devices does not improve the prognosis of patients, but these interventional protective devices can still be applied to patients with heavy plaque loading. Stabilization of plaque, platelet inhibition, and vasodilatation in pharmacotherapy can help prevent CME and reduce coronary microvascular injury [3].

Pyroptosis, as pro-inflammatory programmed cell death, has some characteristics of apoptosis, such as caspase-dependent, cellular DNA damage, and nuclear solidification. In the process of pyroptosis, the inflammatory corpuscle receptors are activated. Through caspase-1 or caspase-11, -4, and − 5, gasdermin D (GSDMD) is sheared, and the N-terminal domain is released freely, forming pores on the cell membrane, and mediating the release of cell contents. In the caspase-1-dependent pathway, activated caspase-1 promotes IL-1β and IL-18 maturation, which are released into the extracellular environment through the pores and amplify the inflammatory response [4, 5].

NOD-like receptor pyrin containing 3 (NLRP3) is an inflammasome of multi-protein complexes. NLRP3 inflammasome is composed of NLRP3, apoptosis-associated speck-like protein containing a CARD (ASC) and pro-caspase-1. ASC is an important adaptor protein of inflammasome, which interacts with NLRP3 through the N-terminal pyrin domain (PYD), and the C-terminal caspase activation and recruitment domain (CARD) can recruit pro-caspase-1 [6, 7]. The activation of NLRP3 mediates the occurrence of pyroptosis and is associated with the occurrence and development of various cardiovascular diseases. It is found that the activation of NLRP3 is involved in the formation of atherosclerosis [8]. NLRP3 in the peripheral blood of patients with acute coronary syndrome is correlated with coronary atherosclerosis, which is positively correlated in severity [9]. In addition, inhibiting the activation of NLRP3 inflammasomes can alleviate myocardial ischemia-reperfusion (I/R) injury and improve cardiac dysfunction induced by isoproterenol by inhibiting cardiomyocyte aging and oxidative stress [10, 11]. In CME, NLRP3 inflammasome activation mediated pyroptosis is associated with myocardial injury [12, 13]. Therefore, regulating NLRP3 inflammasomes may be a potential target for prevention and treatment of cardiovascular diseases.

Adenosine monophosphate-activated protein kinase (AMPK) and silent information regulation T1 (SIRT1) are involved in maintaining cellular energy homeostasis [14]. The AMPK/SIRT1 pathway mediates myocardial fibrosis, cardiac remodeling, and systolic dysfunction in aging, and is involved in reducing myocardial I/R injury and reducing diabetic myocardial microvascular injury [15–18]. A previous study also found that the AMPK pathway mediates myocardial pyroptosis caused by CME [19].

Colchicine is a kind of preparation that can inhibit the polymerization of tubulin and has extensive anti-inflammatory effects. In recent years, there have been relevant clinical studies exploring the effects of colchicine in coronary artery disease, including reduced infarct size under Magnetic Resonance Imaging (MRI) assessment, reduced the risk of ischemic cardiovascular events in patients with myocardial infarction, and improved the morphology of coronary plaques in acute coronary syndrome (ACS) [20–22].

In summary, these studies show the potential benefits of colchicine in coronary artery disease, but the efficacy of colchicine in CME is unclear. Therefore, this study intends to explore the effects of colchicine in myocardial pyroptosis caused by CME and the related mechanism.

## Materials and methods

### Animal modeling and grouping

Sprague-Dawley (SD) rats (weight 250-300 g, male, 8 weeks old) were purchased and housed at the Experimental Animal Center of Guangxi Medical University. The rat CME model was constructed as previously reported [23]. Anesthesia was performed by intraperitoneal injection of pentobarbital sodium (dosage 30–40 mg/kg). After the completion of tracheal intubation and small animal ventilator-assisted respiration in rats, the heart was exposed by opening the chest, and the ascending aorta was isolated and exposed, then the ascending aorta was subsequently clamped while 100 µl of polyethylene microspheres solution (polysciences, USA) containing 8000 microspheres was injected from the apical region. The duration of clamping was about 10 s, followed by closure of the thoracic cavity with layer-by-layer sutures, and removal of the tracheal tube after the rats were resuscitated from anesthesia. The animal experimental design followed the recommended procedures of the National Institutes of Health and was also approved by the Animal Care & Welfare Committee of Guangxi Medical University.

The rats were randomly divided into 4 groups, including the Sham group, CME group, CME + colchicine (CME + COL), CME + colchicine + compound C (an AMPK pathway inhibitor) (CME + COL + CC), and 10 rats in each group. The Sham group was operated as the CME group, but the left ventricle was infused with saline alone. Colchicine was given at 8:00 a.m. by gavage seven days before and on the day of modeling (dosage 0.3 mg/kg, dissolution of sterile water). The dose gradient pre-experiment was performed to determine the dose of colchicine. The setting of the dose gradient was based on other cardiovascular and metabolic disease model studies in combination with the equivalent conversion of drug dose in rat and human body surface area [24–27]. The dose of colchicine 0.3 mg/kg was determined based on the improvement of cardiac function and the reduction of myocardial injury in rats after CME in the gradient dose pre-experiment (Supplemental Figure S1). In the CME + COL + CC group, after colchicine was given seven days before and on the day of modeling, compound C (0.25 mg/kg, dissolution of sterile water) was pushed through the tail vein half an hour before modeling.

### Echocardiography

Cardiac function in rats was assessed by ultrasonic cardiograph (MyLabSix, Esaote, Italy), which measured left ventricular end-diastolic diameter (LVEDd), left ventricular end-systolic diameter (LVESd), left ventricular ejection fraction (LVEF) and left ventricular fractional shortening (LVFS). According to the previous study, the peak of cardiomyocytes scorching injury after the acute phase of CME corresponds to the time point of the most severe impairment of cardiac function at nine hours, so the measurements were performed at nine hours after the rats were modeled [13]. Echocardiograms were performed by an experienced clinical cardiologist, and the results were analyzed based on the average of three cardiac cycles.

### Sample collection and processing

After completion of echocardiography, the rats were injected intraperitoneally with an overdose of sodium pentobarbital, followed by collection of abdominal aortic blood and isolation of the serum for subsequent ELISA testing. The heart was then quickly removed, washed in cold saline, the atrial and auricular portions were removed, then the left ventricle was incised along the long axis of the left ventricle, leaving the apical and middle portions stored at -80° for subsequent quantitative real-time polymerase chain reaction (qRT-PCR) and Western blot analyses. The base of the heart was fixed in 4% paraformaldehyde. After paraffin embedding, the tissues were cut into 4-micron sections for subsequent histopathological staining and immunofluorescence.

### Serum myocardial injury markers

The levels of lactate dehydrogenase (LDH), cardiac troponin T (cTnT), and creatine kinase myocardial band isoenzyme (CK-MB) in rat serum samples were detected by a commercial enzyme-linked immunosorbent assay (ELISA) kit (Bio-Swamp, China) according to the manufacturer’s instructions.

### Hematoxylin and eosin (HE) staining

The 4-micron paraffin sections of the rat ventricle were first dewaxed and rehydrated, followed by hematoxylin and eosin staining, and finally were dehydrated and sealed. An optical microscopy (ZEISS, Axio Imager.A2, Germany, 200 times magnification) was used for observation and analysis.

### Hematoxylin-basic fuchsin-picric acid (HBFP) staining

HBFP staining was used to evaluate myocardial infarction in rats after CME. The 4-micron ventricular myocardial paraffin sections were dewaxed, followed by hematoxylin staining, alkaline fuchsin staining, and differentiating in picric acid-acetone solution. Finally, the sections were dehydrated and sealed. The results were observed through an optical microscope (ZEISS, Axio Imager.A2, Germany, 200 times magnification). Ischemic myocardium and erythrocytes were stained with red, and the normal myocardium was stained with yellow or brownish yellow, then the nuclei were stained with blue black. The measurements of infarct size were performed using the Image J (NIH, MD, USA) software.

### Immunofluorescence staining

We used Immunofluorescence (IF) staining to observe the expression level of NLRP3, caspase-1 p20, and SIRT1 in rat myocardial tissue. Briefly, the myocardial tissue sections were fixed with 4% paraformaldehyde and sealed with 5% BSA after 0.5% Triton X (Solarbio, China) penetration, followed by overnight incubation with NLRP3 (dilution rate 1:200, Abcam, USA), caspase-1 p20 (dilution rate 1:200, Bioss, China) and SIRT1 (dilution rate 1:100, Abcam, USA) primary antibody. The next day, the fluorescent second antibody (dilution rate 1:1000, Abcam, USA) was incubated in a dark room for 1 h. Finally, the nucleus was stained with DAPI. The results were observed with a fluorescence microscope (ZEISS, Axio Imager.A2, Germany, 200 times magnification).

### Quantitative real-time PCR

Total RNA in rat myocardial tissue was extracted by the TRIzol reagent. The concentration and purity of RNA were detected using the NanoDrop 2000 instrument (Thermo Fisher Scientific Inc., USA). RNA reverse transcription to cDNA was performed according to the procedure used in reverse transcription kits (Takara, Japan). The qRT-PCR reaction was performed on an ABI 7500 instrument using SYBR Green I PCR kit (Takara, Japan) reagent. The relative expression of the target genes and the internal reference gene GAPDH was calculated by the 2^−ΔΔCt^ method. A list of the primer sequences is shown in Table 1.

**Table 1:** List of the primer sequences in RT-qPCR

Gene		Primer Sequence
NLRP3	Forward	5′-CAGACCTCCAAGACCACGACTG-3′
	Reverse	5′-CATCCGCAGCCAATGAACAGAG-3′
ASC	Forward	5′-GCTGAGCAGCTGCAAAAGAT-3′
	Reverse	5′-GCAATGAGTGCTTGCCTGTG-3′
IL-1β	Forward	5′-GGGATGATGACGACCTGCTA-3′
	Reverse	5′-TGTCGTTGCTTGTCTCTCCT-3′
GAPDH	Forward	5′-GAAGGTCGGTGTGAACGGAT-3′
	Reverse	5′-CCCATTTGATGTTAGCGGGAT-3′

### Western blotting

The extraction of total myocardial tissue proteins was completed using RIPA lysis buffer (Solarbio, China), followed by the determination of protein concentration using a BCA kit (Solarbio, China). The proteins were denatured at high temperature, separated by SDS-PAGE electrophoresis, and then transferred to PVDF membranes (Millipore, USA). After completing the closure (60 min) at room temperature using non-fat milk, the proteins were incubated overnight at 4 ℃ with SIRT1 (dilution rate 1:1000, Abcam, USA), AMPK (dilution rate 1:1000, Abcam, USA), p-AMPK (dilution rate 1:1000, Abcam, USA), NLRP3 (dilution rate 1:1000, proteintech, China), GSDMD-N (dilution rate 1:1000, Cell Signaling Technology, USA), ASC (dilution rate 1:1000, Abcam, USA), IL-18 (dilution rate 1:1000, Abcam, USA), caspase-1 p20 (dilution rate 1:1000, Bioss, China), IL-1β (dilution rate 1:1000, Abcam, USA), and GAPDH (dilution rate 1:10000, Abcam, USA) primary antibodies. The proteins were incubated with secondary antibodies (dilution rate 1:10000, Santa Cruz Biotechnology, USA) at room temperature for 1 h the next day, and the protein bands were developed under the ultrasensitive automatic chemiluminescence imaging analysis system (FluorchemM, Protein Simple, USA). The detection of relative protein expression was completed using Image J (NIH, MD, USA) software.

### Statistical analysis

The data of this study were analyzed using SPSS software (26.0 IBM, USA). The data were presented as mean ± standard deviation (SD). Comparisons involving multiple groups were performed using One-way analysis of variance (ANOVA) followed by a post hoc test. P value < 0.05 was set as the threshold for statistical difference.

## Results

### Colchicine alleviated the cardiac dysfunction of the rat suffering from CME

**Fig. 1:**
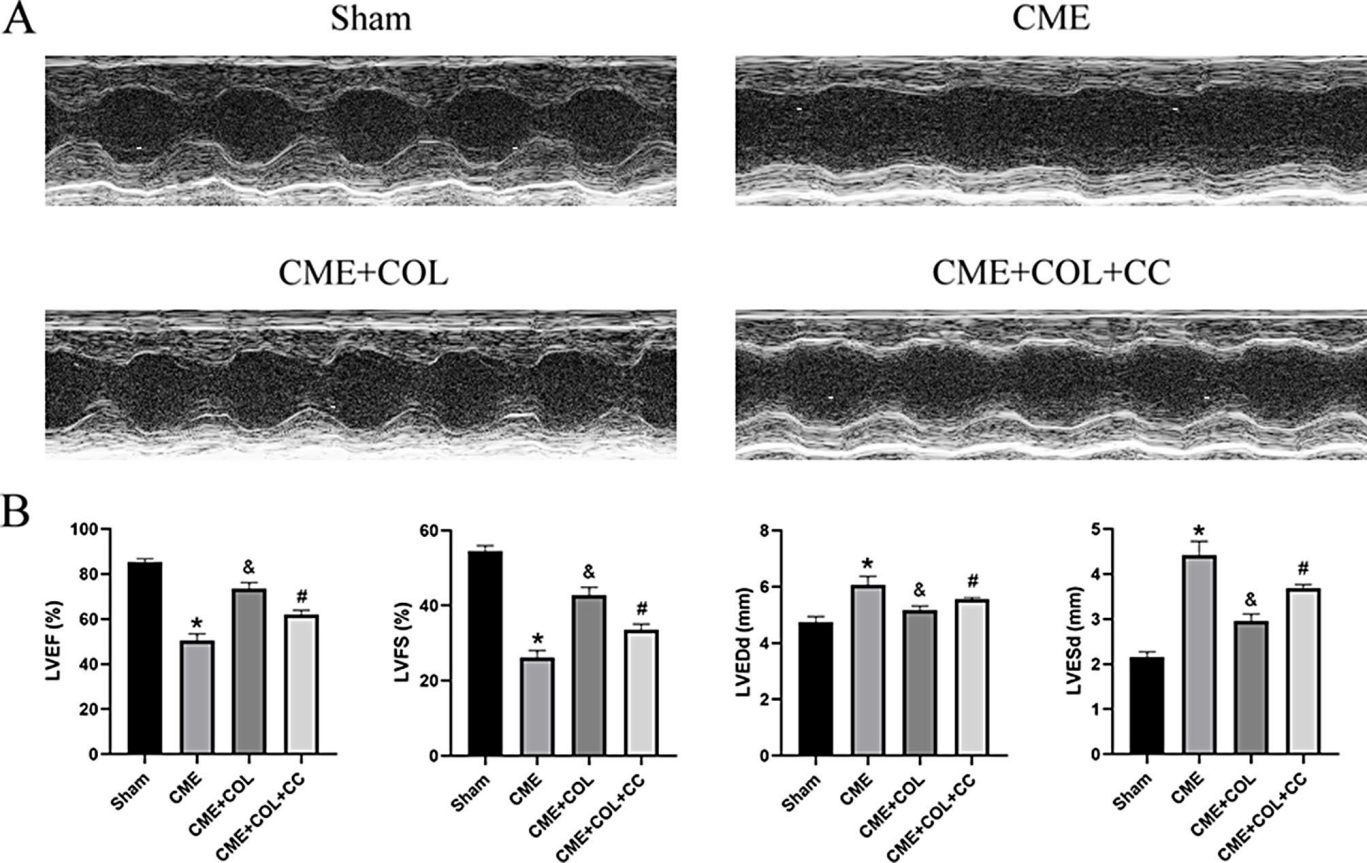
Colchicine improves cardiac dysfunction caused by CME. (**A**) The representative echocardiography in each group. (**B**) The measurement results of left ventricular end-diastolic diameter (LVEDd), left ventricular endystolic diameter (LVESd), left ventricular ejection fraction (LVEF), and left ventricular fractional shortening (LVFS) (n = 5 per group). ^*^p<0.05 versus Sham group; ^&^p<0.05 versus CME group; ^#^p<0.05 versus CME + COL group

The changes of cardiac function in rats suffering from CME were evaluated by echocardiography. The results showed that the LVEF and LVFS of the CME group were significantly lower than those in the Sham group, accompanied by an increase in LVEDd and LVESd. After colchicine pretreatment, the cardiac function of rats was improved, which was manifested by the increase of LVEF and LVFS, and the decrease of LVEDd and LVESd. However, the improvement of cardiac function by colchicine pretreatment was reversed by compound C co-treatment (Fig. 1).

### Colchicine attenuates myocardial injury in a CME model

**Fig. 2:**
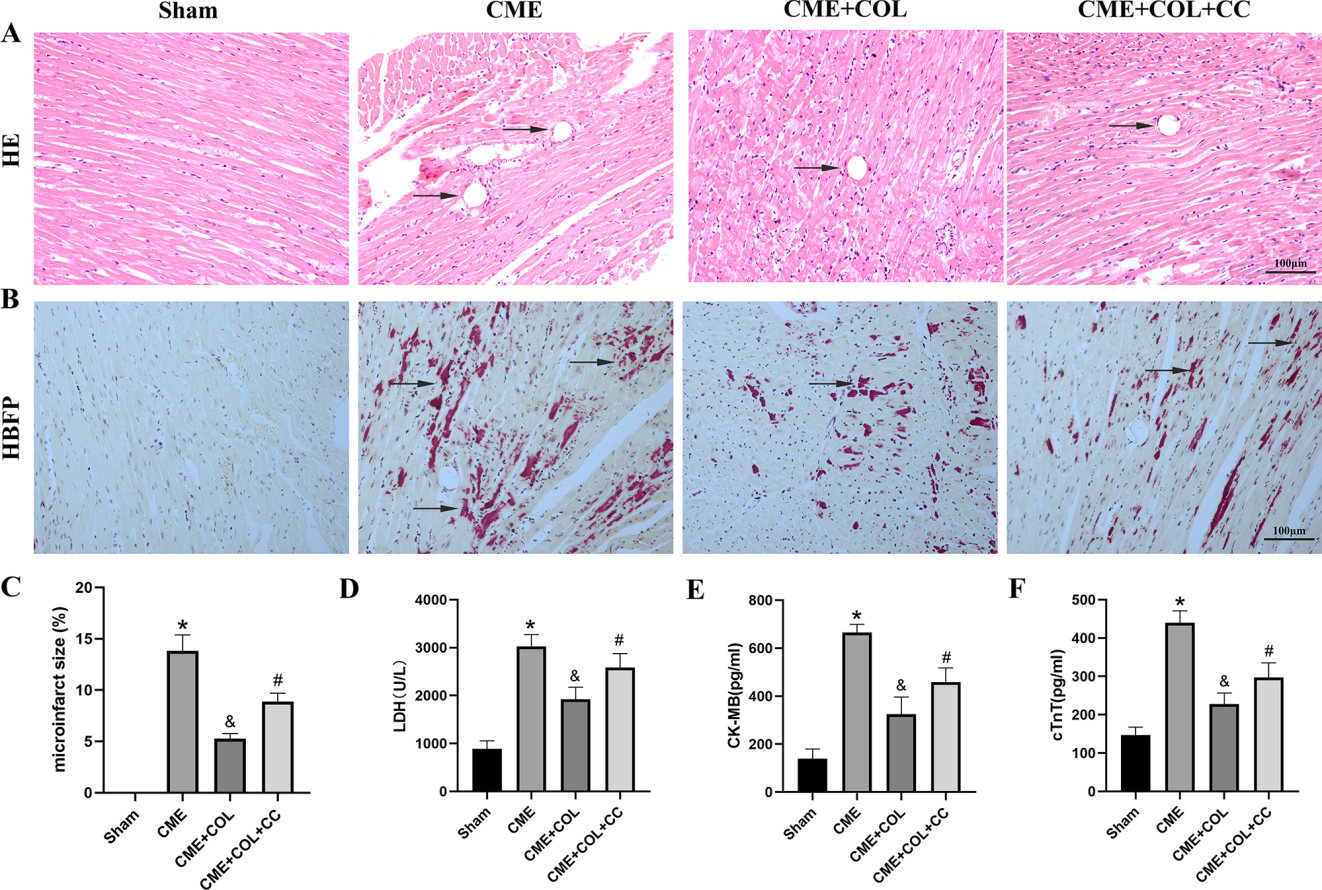
Colchicine attenuates myocardial injury induced by CME. (**A**) Representative morphological images of HE staining of rat hearts in each group (Magnification,×200; Scale bar = 100 μm). Black arrows indicate the microspheres; (**B**) Representative morphological images of HBFP staining of rat hearts in each group (n = 5) (Magnification,×200; Scale bar = 100 μm). Black arrows indicate the microinfarct area. (**C**) Quantitative examination of microinfarct area for each group. (**D**-**F**) The levels of serum LDH, CK-MB, and cTnT in each group (n = 5). ^*^p<0.05 versus Sham group; ^&^p<0.05 versus CME group; ^#^p<0.05 versus CME + COL group

In the morphological images of HE staining, the myocardial fibers were clear, intact, and neatly arranged, without tissue edema and inflammatory cell infiltration in the Sham group (Fig. 2A). The cardiomyocytes in the infarcted area caused by the microspheres in the CME group were disordered, distorted, hypertrophic, and accompanied by obvious inflammatory cell infiltration (Fig. 2A). In addition, we observed multiple microinfarcts of myocardial tissue caused by microspheres in the CME group by HBFP staining (Fig. 2B). Notably, the degeneration, edema, and inflammatory cell infiltration of cardiomyocytes were reduced in CME + COL group in HE staining. In HBFP staining, the microinfarcts in the CME + COL group were significantly lower than those in the CME group. Similarly, in the CME + COL + CC group, the benefits of colchicine pretreatment in myocardial injury were partially reversed (Fig. 2B and C).

### Colchicine improves serum myocardial injury markers

The changes of serum myocardial injury markers were shown in Fig. 2D, E and F. Serum LDH, CK-MB, and cTnT in the CME group were significantly increased compared with the Sham group, indicating myocardial injury suffering from CME. In the CME + COL group, we observed that serum LDH, CK-MB, and cTnT were significantly decreased, showing an improvement in myocardial injury. After adding CC, serum LDH, CK-MB, and cTnT increased compared with the CME + COL group, which partially reversed the improvement of myocardial injury.

### Colchicine inhibited NLRP3 inflammasome activation and improved myocardial pyroptosis in CME

**Fig. 3:**
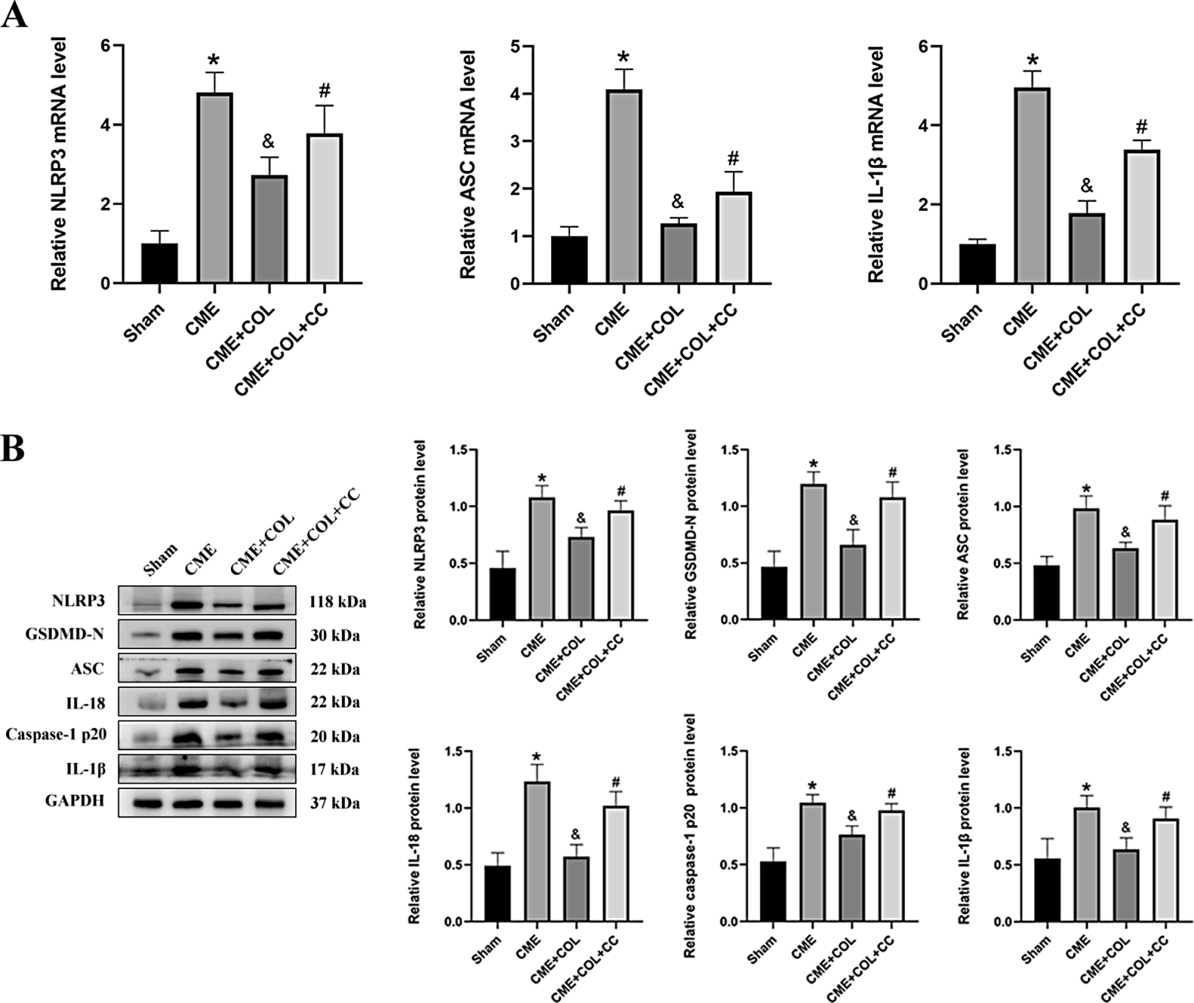
Colchicine ameliorates myocardial pyroptosis induced by CME. (**A**) The changes of mRNA expression levels in NLRP3, ASC, and IL-1β for each group (n = 5). (**B**) The changes of protein expression levels in NLRP3, GSDMD-N, ASC, IL-18, IL-1β, and caspase 1 p20 for each group. n = 4 for each group. The blots in Fig. 3B were from different gels. The original western blot images were provided in the supplementary file. ^*^p<0.05 versus Sham group; ^&^p<0.05 versus CME group; ^#^p<0.05 versus CME + COL group

**Fig. 4:**
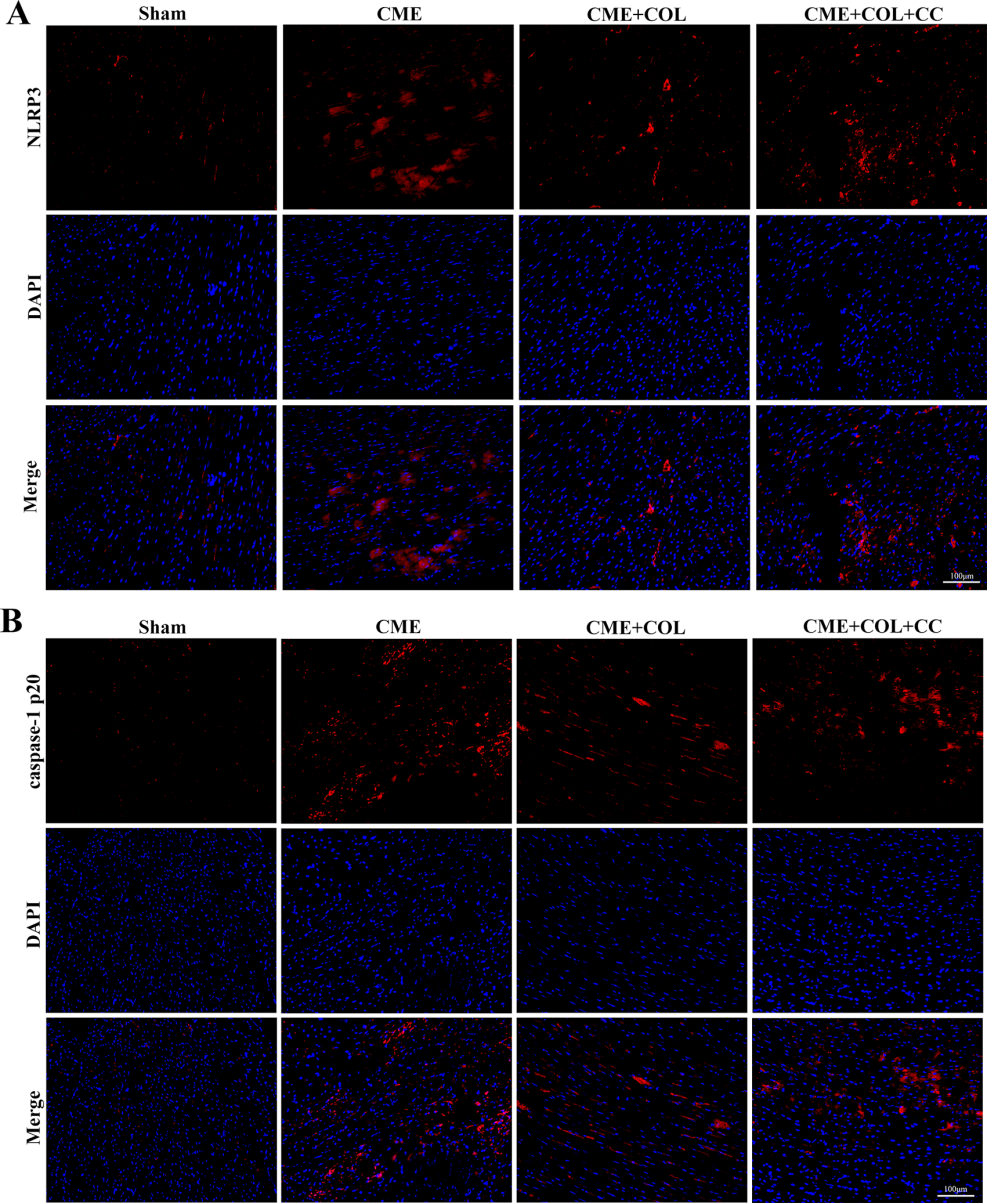
The immunofluorescence images of NLRP3 and caspase-1 p20 in rat myocardial tissue for each group (n = 4). (**A**) Representative immunofluorescence images of NLRP3. (**B**) Representative immunofluorescence images of caspase-1 p20. Magnification,×200; Scale bar = 100 μm

As shown in Fig. 3, the mRNA levels of NLRP3, ASC, and IL-1β in the CME group were significantly increased compared with the Sham group, and the corresponding NLRP3, ASC, IL-1β, IL-18, GSDMD-N, and caspase-1 p20 protein expression levels were also significantly increased in the CME group. Notably, the mRNA and protein expression levels of NLRP3, ASC, IL-1β, IL-18, GSDMD-N, and caspase-1 p20 were significantly decreased in the CME + COL group. However, after co-treatment with CC, the decreased mRNA and protein expression levels were reversed. Correspondingly, the same trends of protein expression levels in NLRP3 and caspase-1 p20 were observed in IF (Fig. 4A and B).

### Colchicine inhibits cardiomyocyte pyroptosis through the AMPK/SIRT1/NLRP3 signaling pathway

**Fig. 5:**
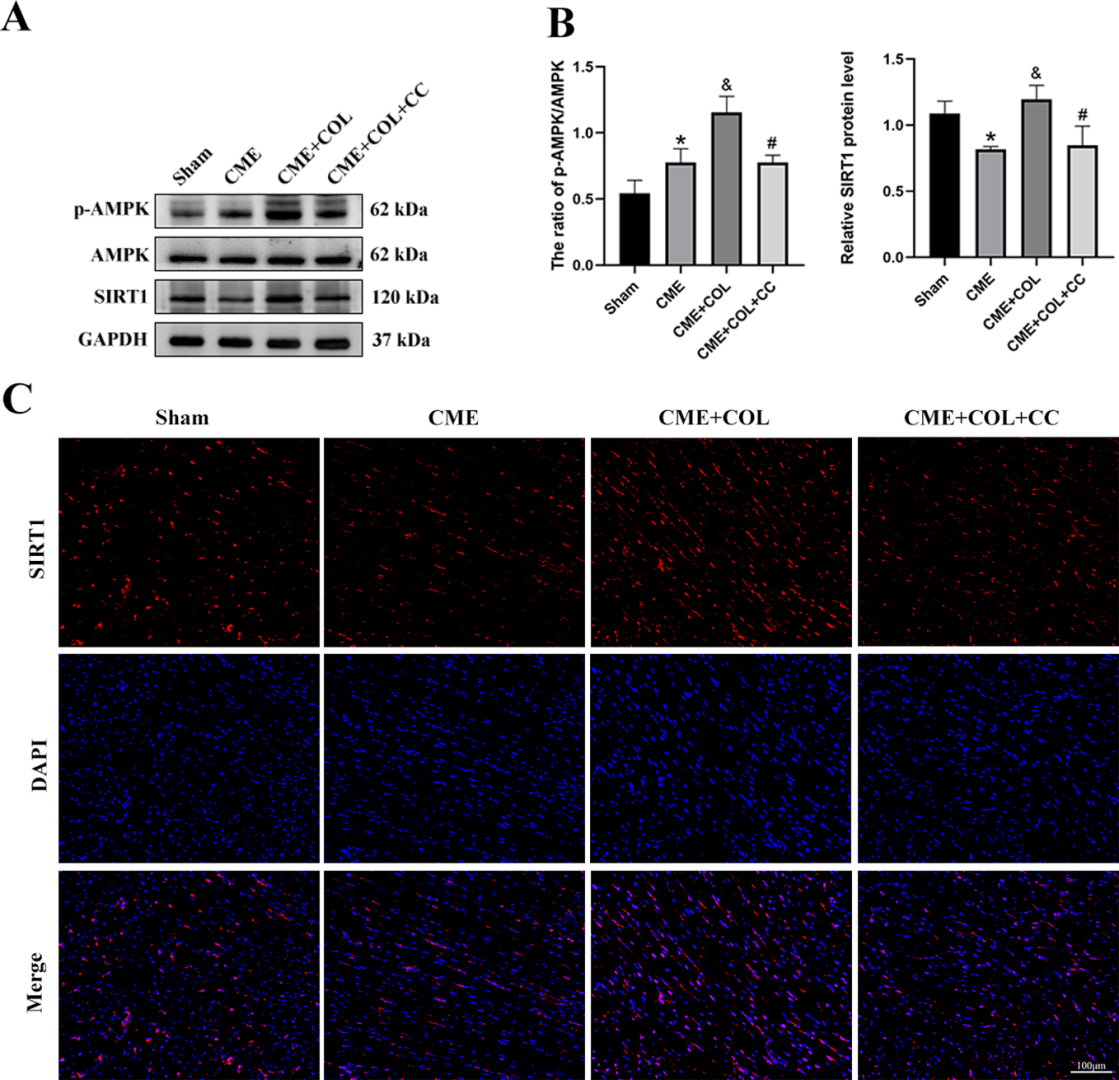
Colchicine inhibits pyroptosis through the AMPK / SIRT1 / NLRP3 signaling pathway. (**A**-**B**) The changes of protein expression levels in p-AMPK, AMPK, and SIRT1 for each group (n = 4). The blots in Fig. 5A were from different gels. The original western blot images were provided in the supplementary file. (**C**) The immunofluorescence images of SIRT1 in rat myocardial tissue for each group (n = 4). ^*^p<0.05 versus Sham group; ^&^p<0.05 versus CME group; ^#^p<0.05 versus CME + COL group

As shown in Fig. 5A, we observed that the protein expression level of total AMPK did not change significantly in the four groups, while the protein expression level of phosphorylated AMPK was significantly increased in the colchicine pretreatment group. SIRT1 was significantly decreased in the CME group compared with the Sham group. After colchicine pretreatment, SIRT1 was significantly increased. However, when CC was co-treated, the increased expression level of phosphorylated AMPK and SIRT1 was reversed. In the results of IF in SIRT1 (Fig. 5C), we observed the results consistent with Western blotting (Fig. 5B).

## Discussion

This study successfully constructed a rat CME model, and the results showed that under CME injury, the activation of NLRP3 inflammasome was induced, which further led to the occurrence of myocardial pyroptosis. Pretreatment with colchicine can reduce myocardial pyroptosis by inhibiting the activation of NLRP3 inflammasome, thus reducing the damage of cardiomyocytes and improving the cardiac function of rats. In terms of mechanism, we found that the AMPK/SIRT1/NLRP3 signaling pathway is involved in colchicine-mediated protection of myocardial pyroptosis induced by CME.

The pathophysiology of CME involves the activation and damage of inflammation, and anti-inflammatory therapy plays a role in reducing cardiomyocyte damage after CME [28, 29]. Pyroptosis, as a pro-inflammatory programmed cell death, has been found to mediate the occurrence and development of various cardiovascular diseases. Piamsiri et al. found that pyroptosis played a major role in myocardial injury and cardiac dysfunction after myocardial infarction by comparing the programmed cell death modes such as apoptosis, necrotic apoptosis, pyroptosis, and ferroptosis in rats after myocardial infarction [30]. Liu et al. found that pyroptosis was activated in mice with acute myocardial infarction, and the use of pyroptosis inhibitors could significantly reduce the infarct area and improve cardiac function [31]. Kang et al. found that myocardial I/R injury induced myocardial pyroptosis. LncRNA Rian could promote the transcription of downstream gene CCND1 by competitively binding miR-17-5p, inhibit myocardial pyroptosis, and improve myocardial I/R injury in mice [32]. Shi et al. found that GSDMD-mediated myocardial pyroptosis is involved in the process of myocardial I/R injury. Inhibiting GSDMD could reduce myocardial pyroptosis and improve myocardial injury [33]. Pyroptosis was also involved in myocardial damage caused by CME. Inhibiting myocardial pyroptosis could improve myocardial damage caused by CME and improve cardiac function of rats [13, 19, 34]. In this study, we confirmed that CME induced cardiomyocyte pyroptosis by means of molecular biology. After pretreatment with colchicine, the expression levels of mRNA and protein related to pyroptosis were down-regulated, indicating the potential role of colchicine in improving CME-induced cardiomyocyte pyroptosis.

Colchicine, for its anti-inflammatory action usually applied in acute attacks of gouty arthritis, was further studied for the effects in potential anti-fibrotic and anti-tumor [35, 36]. Recent studies have explored the application of colchicine in various cardiovascular diseases. Shen et al. found that colchicine reduced inflammation, inhibited NLRP3 inflammasome activation, and improved cardiac dysfunction and myocardial fibrosis in rats with heart failure with preserved ejection fraction [25]. Fujisue et al. found that the treatment of colchicine in myocardial infarction for 4 weeks could improve LVEDd in cardiac function and ejection fraction, reduce heart failure, and significantly improve survival in the infarcted mice. Mechanistically, colchicine was found to reduce the release of pro-inflammatory factors and the activation of NLRP3 inflammasome in the infarcted myocardium of mice 24 h after infarction [37]. Li et al. found that colchicine attenuated cardiac remodeling and improved cardiac function and survival in mice with myocardial infarction by inhibiting the formation of neutrophils extracellular traps and inflammatory responses [38]. In addition, colchicine can improve myocardial fibrosis and myocardial remodeling in animal models of atrial fibrillation [39, 40]. Notably, in recent years the clinical application of colchicine in the field of coronary artery disease has been more widely recognized. Clinical studies have found that colchicine has potential benefits in the secondary prevention of coronary artery disease and the stabilization of coronary plaque [21, 41–43]. The latest studies have shown that it was feasible to give P2Y12 inhibitor monotherapy combined with low-dose colchicine on the day after percutaneous coronary intervention in patients with ACS [44]. In this study, based on the anti-inflammatory and anti-inflammasome effects of colchicine, we aimed to explore its application in CME. We found that colchicine pretreatment inhibited the activation of NLRP3 inflammasome and reduced myocardial pyroptosis, thus reducing myocardial injury and improving cardiac dysfunction in rats with CME. This also opens up a new vision for the use of colchicine in CME.

AMPK and SIRT1 are involved in maintaining cellular energy homeostasis. Studies have shown that the AMPK / SIRT1 pathway mediates the protective effects of various cardiovascular diseases. Potenza et al. found that adiponectin pretreatment could mediate the protective effect of myocardial I/R injury in mice through the AMPK / SIRT1 pathway [17]. Similarly, Liu et al. found that arctigenin could reduce oxidative stress and inflammatory response in rats with acute myocardial I/R injury through the AMPK / SIRT1 pathway, reducing cardiomyocyte apoptosis, thereby improving cardiac function and reducing myocardial infarction area [45]. Song et al. found that the decreased expression of p-AMPK and SIRT1 was associated with the progression of myocardial fibrosis in aged rats. Up-regulation of AMPK and SIRT1 may be a potential therapeutic strategy to improve aging-related myocardial fibrosis and cardiac remodeling [15]. Wang et al. found that melatonin could improve oxidative stress and apoptosis damage of cardiac microvascular endothelial cells induced by high glucose by regulating the activity of the AMPK / SIRT1 signaling pathway, and improve cardiac dysfunction induced by streptozotocin in mice [18]. Oxidative stress injury and inflammatory response are important factors in CME-induced myocardial injury. Previous studies have shown that increased production of reactive oxygen species (ROS) and activation of the NF-κB pathway in CME can mediate the activation of NLRP3 inflammasome [12, 46]. SIRT1 is involved in the regulation of redox and inflammation. Studies have shown that SIRT1 can inhibit ROS production and NF-κB pathway activation, thereby inhibiting the activation of NLRP3 inflammasome and reducing pyroptosis [47, 48]. In this study, p-AMPK was slightly increased in CME, which may be an endogenous compensatory protection mechanism in rats [19], while SIRT1 expression was significantly down-regulated in CME. When colchicine was pre-treated, AMPK phosphorylation was promoted, and SIRT1 expression was significantly up-regulated, thereby inhibiting NLRP3 inflammasome, reducing pyroptosis, and improving CME-induced myocardial injury. After the addition of AMPK inhibitor compound C, the protective effect of colchicine was inhibited, and the expression of p-AMPK and SIRT1 was inhibited, which further indicated that the AMPK / SIRT1 / NLRP3 pathway mediated the cardioprotective effect of colchicine pretreatment on CME.

This study also has some limitations. First, the CME model in this study was constructed using polyethylene microspheres to cause physical obstruction, which cannot fully simulate the pathophysiological changes of CME due to atheromatous plaque rupture in the real world. Secondly, this study evaluated the protective effect of colchicine pretreatment on the acute phase of CME, while the therapeutic effects of colchicine in the middle and long term after CME are currently unknown and still need further study. Third, this study investigated the effects of colchicine on CME in vivo in the rat, but no in vitro simulation experiments were completed in vitro using a primary cardiomyocyte model.

## Conclusion

In summary, colchicine inhibits the activation of NLRP3 inflammasome through the AMPK/SIRT1/NLRP3 signaling pathway, and inhibits CME-mediated myocardial pyroptosis, thereby alleviating myocardial injury and improving cardiac function. This study provides a theoretical basis for the preventive effect of colchicine on myocardial injury caused by CME and further therapeutic research in the future.

### Electronic supplementary material

Below is the link to the electronic supplementary material.

Supplementary Material 1

## Data Availability

The data of this study will be provided by the corresponding author according to reasonable requirements.
