# Supplementary material for: Colchicine ameliorates myocardial injury induced by coronary microembolization through suppressing pyroptosis via the AMPK/SIRT1/NLRP3 signaling pathway
Source: BMC Cardiovasc Disord. 2024 Jan 3;24:23. doi: 10.1186/s12872-023-03697-8 (PMC10765930; doi:10.1186/s12872-023-03697-8)
Supplement: Supplementary file 1 — Supplementary Material 1 [file 12872_2023_3697_MOESM1_ESM.pdf]

The original images of the representative western blot bands in Fig.3B

NLRP3

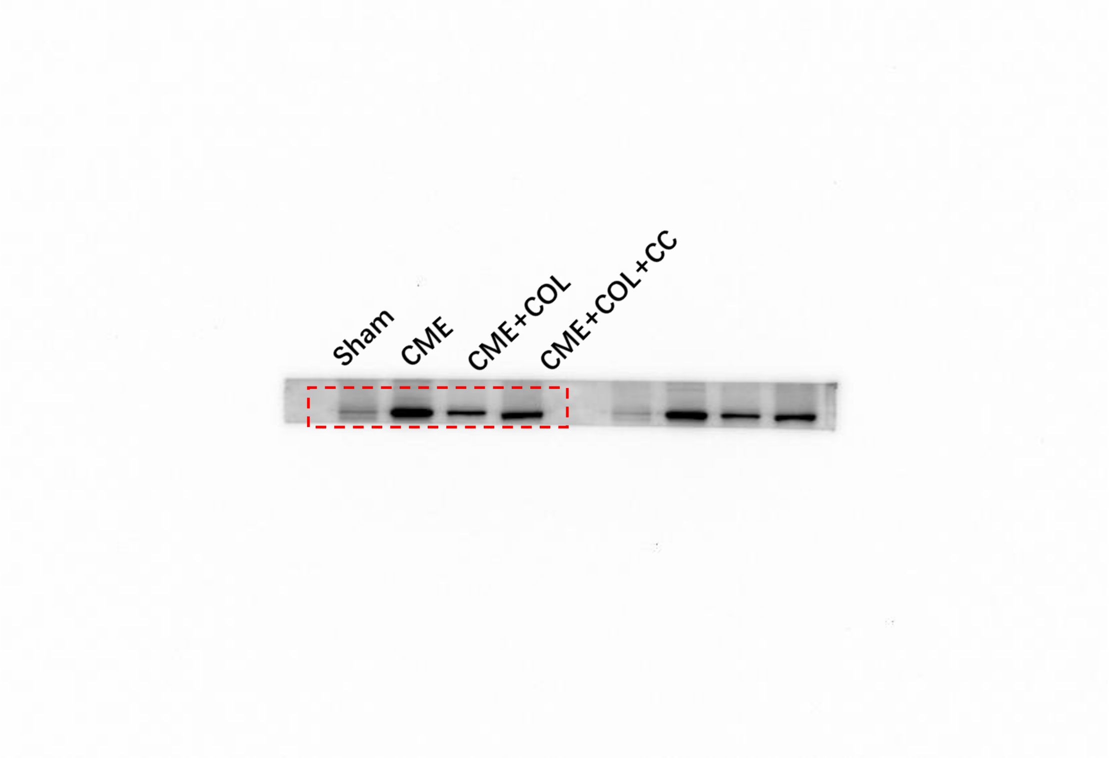

GSDMD-N

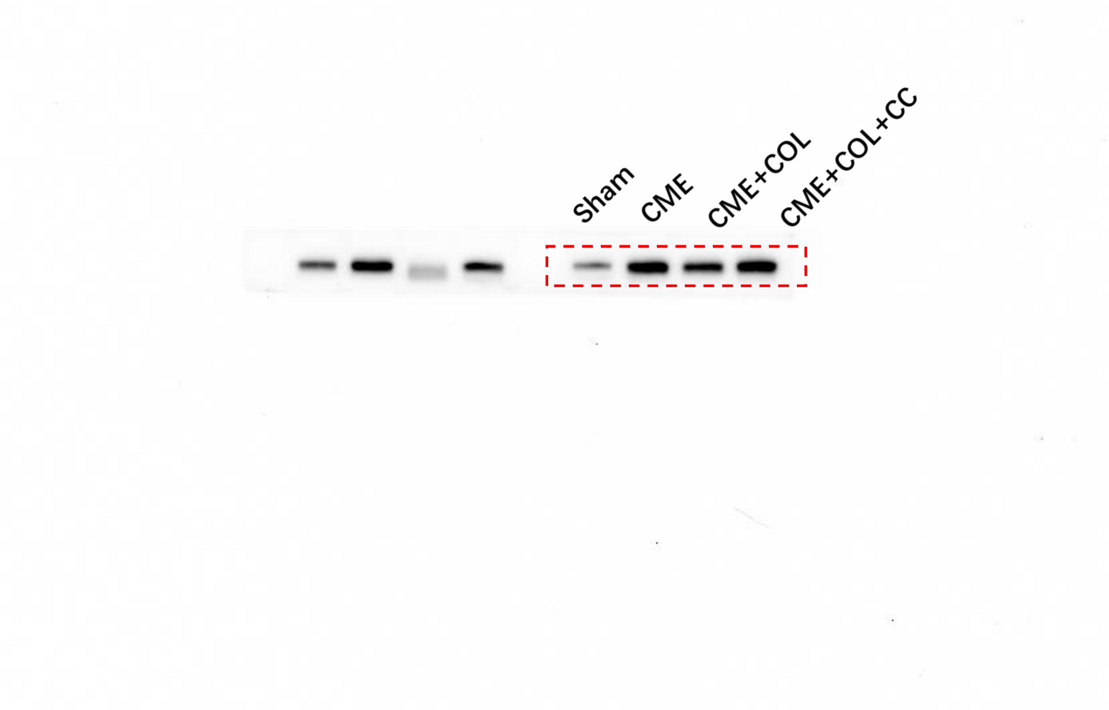

ASC

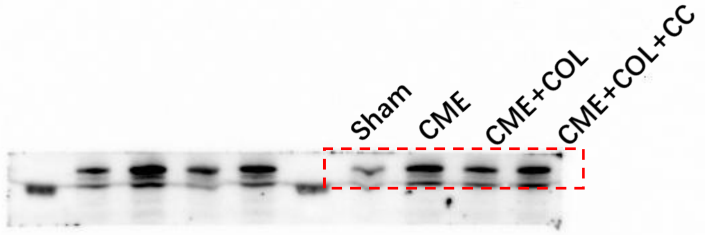

IL-18

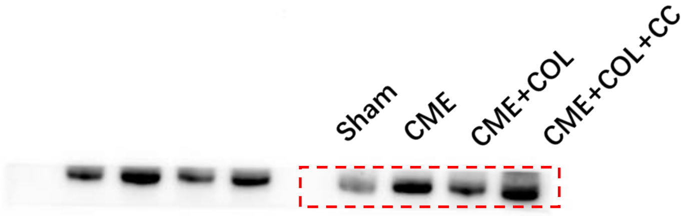

Caspase-1 p20

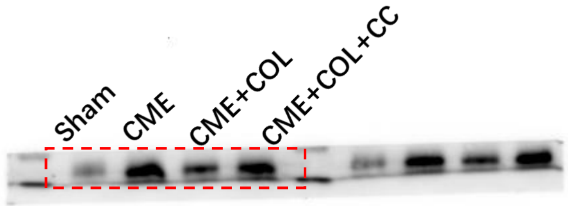

IL-1β

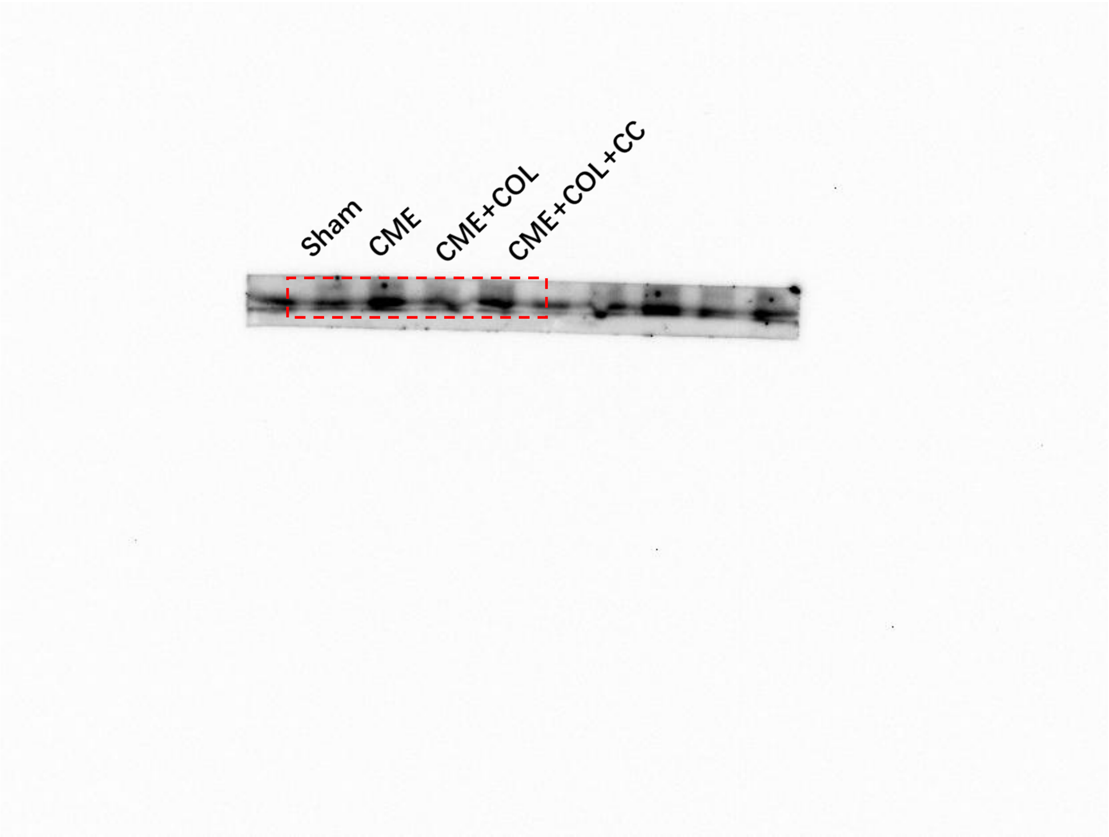

GAPDH

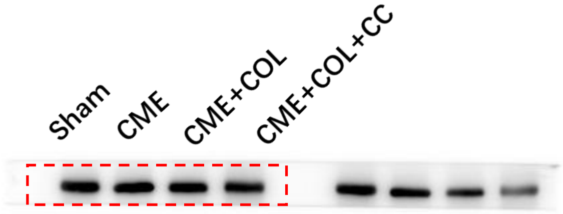

The original images of the representative western blot bands in Fig.5A

P-AMPK

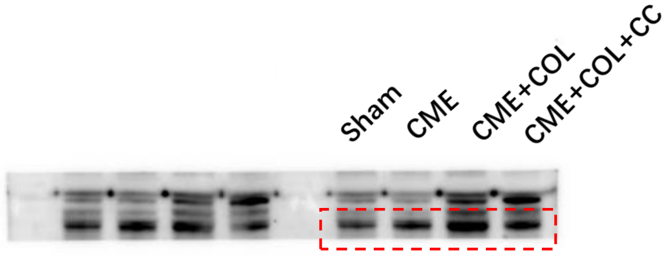

AMPK

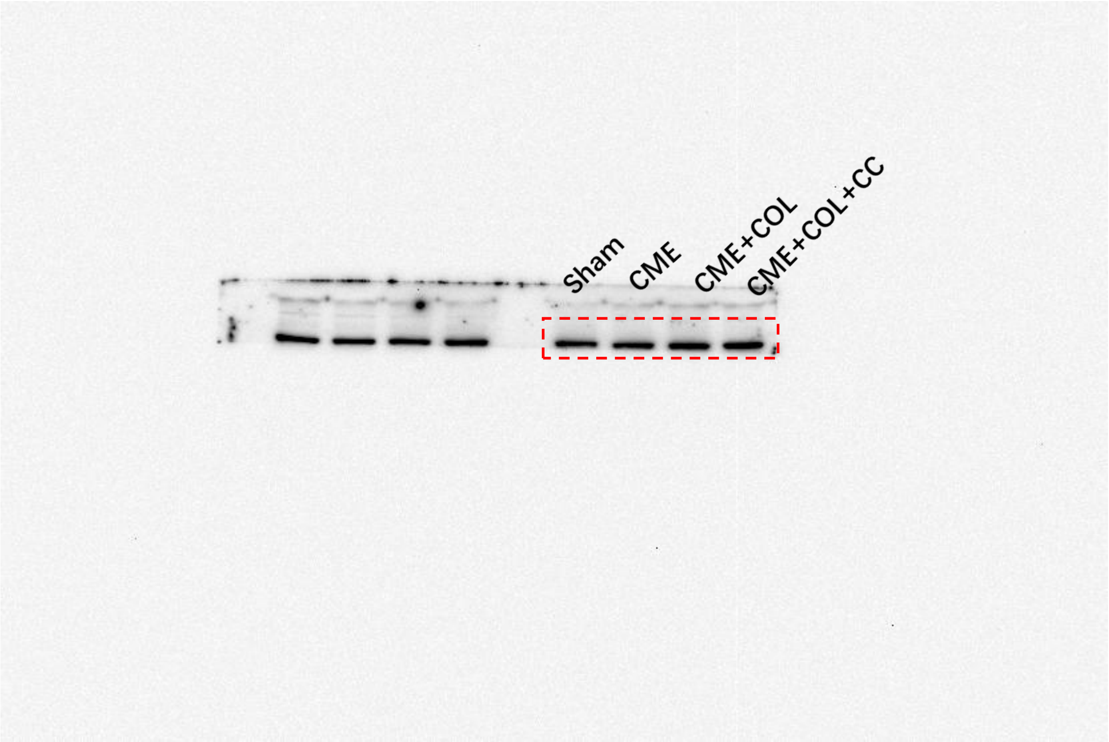

SIRT1

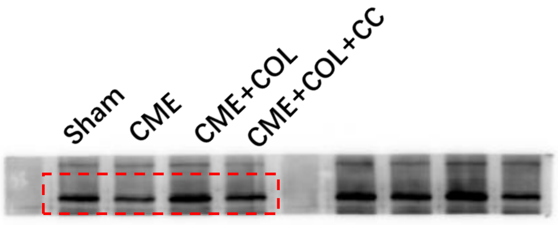

GAPDH

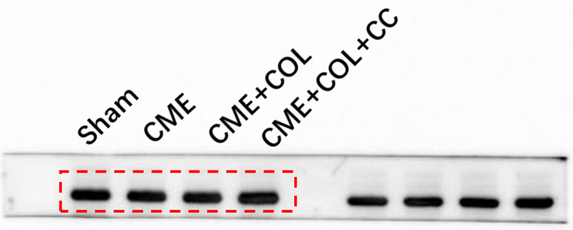

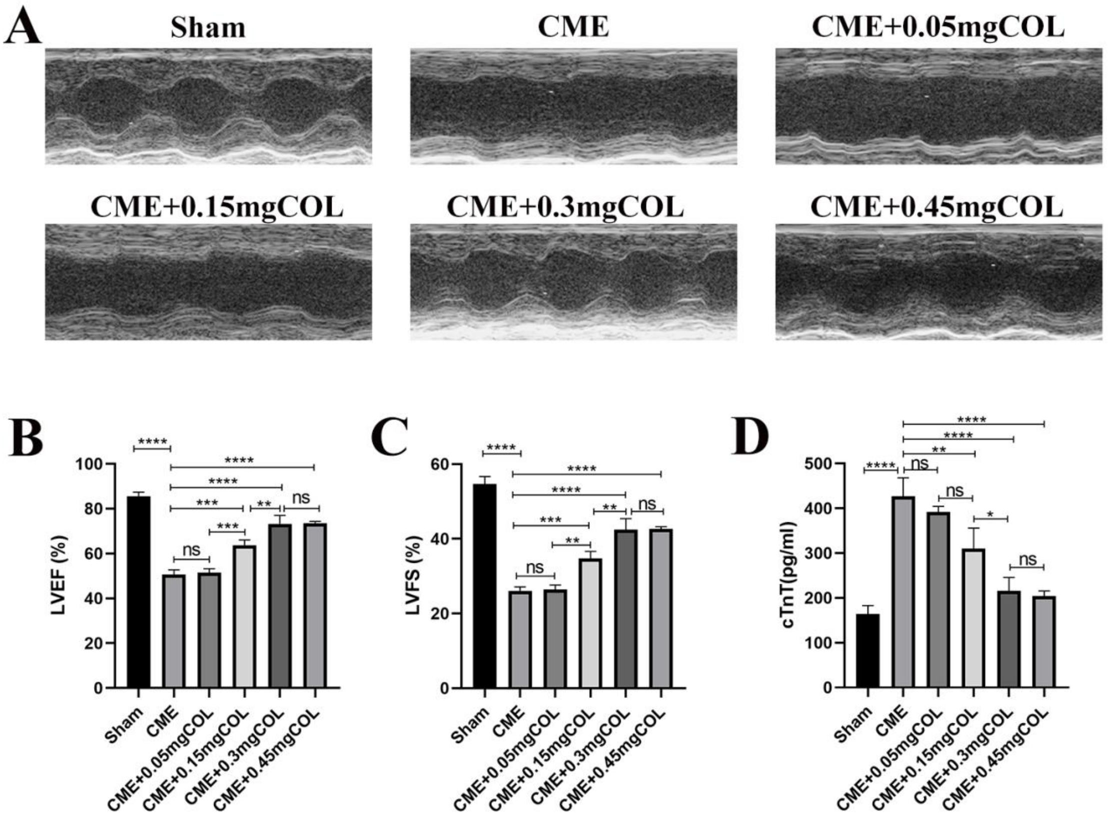

Figure S1. Effects of different doses of colchicine on cardiac function and myocardial injury induced by coronary microembolization in rats. (A) The representative echocardiography in each group. (B-C) The measurement results of left ventricular ejection fraction (LVEF) and left ventricular fractional shortening (LVFS) (n=3 per group). (D) The levels of serum cTnT in each group (n=3). *p < 0.05, **p < 0.01, ***p < 0.001, ****p < 0.0001, ns=no significance.
